# Slight thermal stress exerts genetic diversity selection at coral (*Acropora digitifera*) larval stages

**DOI:** 10.1186/s12864-024-11194-1

**Published:** 2025-01-14

**Authors:** Cristiana Manullang, Nozomi Hanahara, Ariyo Imanuel Tarigan, Yuko Abe, Mao Furukawa, Masaya Morita

**Affiliations:** Sesoko Marine Station, Tropical Biosphere Research Center, University of the Ryukyus, 3422 Sesoko, Motobu, Okinawa, 905-0227 Japan

**Keywords:** Heat stress, Coral larvae, Acropora digitifera, Selective pressure, Tajima's D, Positive selection

## Abstract

**Background:**

Rising seawater temperatures increasingly threaten coral reefs. The ability of coral larvae to withstand heat is crucial for maintaining reef ecosystems. Although several studies have investigated coral larvae’s genetic responses to thermal stress, most relied on pooled sample sequencing, which provides population-level insights but may mask individual genotype variability. This study uses individual larval sequencing to investigate genotype-specific responses to heat stress and the selective pressures shaping their genomes, offering finer resolution and deeper insights.

**Results:**

This study investigates the larval response to heat stress before acquiring symbiotic algae, aiming to elucidate the relationship between coral genetic diversity and heat stress. Larvae sourced from eight *Acropora digitifera* colonies were subjected to ambient temperature (28 °C) and heat conditions (31 °C). The impact of heat stress on larval genetic diversity was assessed through sequencing. While overall genetic diversity, represented by π, did not significantly differ between the control and heat-exposed groups, Tajima’s D differed, indicating different selective pressures in each group. The genomic regions under higher and lower Tajima’s D were not broadly shared among control and head conditions, implying that selective pressures operated in distinctive manners. Many larval protein-coding sequences were identified in this genomic region, and the codon evolution of many of these genes showed signs of positive selection. These results highlight the complex selective pressures on coral larvae under different temperatures. The genes showing signs of positive selection in response to heat stress may have also been influenced by historical temperature fluctuations, as suggested by their association with loci identified during Acroporid speciation. These loci under codon-level positive selection during speciation highlight the potential role of genetic diversity in shaping adaptation to environmental changes over evolutionary timescales.

**Conclusion:**

These findings underscore the significance of genetic diversity in coral reproduction for maintaining reef ecosystems. They also indicate that even minor heat stress can exert significant selective pressure, potentially leading to profound implications for coral reef ecosystems. This research is crucial for understanding the impact of rising seawater temperatures on coral reefs.

**Supplementary Information:**

The online version contains supplementary material available at 10.1186/s12864-024-11194-1.

## Introduction

Coral reef ecosystems are rapidly declining owing to increasing mass bleaching [1, 2]. Larval recruitment following sexual reproduction is a prerequisite for coral reef recovery and maintenance [3]. However, successful larvae recruitment following sexual reproduction is also decreasing [4]. Many reef-building corals exhibit broadcast spawning, generating new larvae from many conspecific colonies. The genomic diversity of the larvae generated from synchronous spawning may be associated with adaptation to future climate change [5, 6]. Initially, these larvae do not have symbiotic algae and rely on their lipid reserves until they acquire symbiotic algae [7–9]. Once symbiotic relationships are established, the larvae receive photosynthetically derived nutrients from the algae, which support their metabolism, growth, and survival. The larvae then disperse and eventually settle [9, 10]. This study focuses on larvae that have not yet acquired symbionts, allowing us to isolate the effects of heat stress on the coral host’s genomic response without the influence of symbiotic algae.

Before establishing a symbiotic relationship, the larval stage reflects some genetic characteristics of the parents, including heat tolerance, although environmental factors, maternal effects, and epigenetic modifications could influence heritability [11]. Previous studies have shown that thermal tolerance is heritable from adults to offspring, but a combination of genetics, environmental history, and other factors shapes this heritability [12–14]. Therefore, the genetic diversity of recently spawned coral larvae is predicted to be associated with their survival under fluctuating water temperatures [6, 14]. Additionally, the water temperature influences the distance of larval dispersal [15–17].

Although several studies have explored the genetic responses of coral larvae to thermal stress [6, 12], most have relied on pooled sample sequencing, providing population-level insights but potentially masking individual genotype variability. Our study, by sequencing individual larvae, allows us to investigate genotype-specific responses to high water temperatures (> 32 °C) and examine the degree of selection across the genomes of stressed and control larvae more precisely. This approach allows for a deeper understanding of how specific genotypes may survive under extreme thermal conditions, which remains underexplored at the individual level.

Genetic diversity is associated with population resilience, as higher genetic variability can enhance its ability to adapt to environmental stressors [18, 19]. However, the relationship between genetic diversity and adaptive potential across populations remains complex and not fully understood. Some modeling studies have suggested that locally adapted populations may retain higher genetic diversity due to selective pressures acting on specific traits under fluctuating conditions [13, 20], but empirical evidence for this is still emerging. The local adaptation of corals implies that genetic diversity is essential for adaptation [21–23]. Our study aims to contribute to this understanding by examining changes in the frequency of specific genotypes under control and heat stress conditions, providing insights into the potential role of genetic diversity in adaptation to thermal stress.

Corals have experienced successive population declines, leading to genetic bottlenecks [21, 24, 25]. Although bottlenecks in heavy bleached populations have been predicted, a heterozygosity-based genetic perspective shows that their genetic diversity tends to recover within a short period [25, 26]. Gene flow has been observed among long-distance metapopulations during reef recovery [18, 26, 27], and while it can disrupt natural selection and local adaptation, it may also facilitate adaptive gene introgression, which enhances resilience to environmental changes. This potential for rapid adaptation in coral populations provides hope for coral reef recovery. Therefore, elucidating changes in genetic diversity at the larval stages could help us understand how larvae adapt to warm conditions and how synchronous spawning among conspecific colonies influences the maintenance or alteration of this diversity for higher fitness.

Historically, coral reefs have undergone considerable environmental changes, including fluctuations in sea temperature [28, 29], which impose varying selective pressures on coral populations [29–31]. These historical selective pressures, along with other forces such as mutations, genetic drift, and gene flow, have shaped the genetic diversity observed in contemporary coral populations [18, 21]. While this may contribute to their adaptive potential [23, 31, 32], it is also possible that selection in specific environments can reduce genetic diversity, potentially limiting the population’s ability to adapt to other pressures. To address these questions, our study investigates the historical selection of candidate genes during speciation, focusing on how these selective forces may have influenced genetic diversity and adaptive potential in coral populations. By exploring these evolutionary processes, we aim to understand better how past selective pressures have shaped the resilience of coral populations to environmental changes.

In this study, we combined genome-wide sequencing of individual coral larvae under both ambient and elevated temperature conditions with proteomic analysis conducted under ambient conditions to identify the proteins present in the larvae. Although the proteomic analysis was performed without heat stress, this approach allowed us to establish a baseline understanding of the protein composition in the larvae, which can inform future studies linking genotype and phenotype under different environmental conditions, including heat stress. This methodological innovation offers a comprehensive understanding of how genetic diversity and selective pressures shape the adaptive potential of coral larvae. However, this perspective has not been explored in previous studies. In this study, we aimed to investigate the impact of heat stress on genetic diversity and selective pressure in coral larvae, specifically examining how short-term exposure to elevated temperatures can influence genetic variation and the occurrence of positive or negative selection, as indicated by Tajima’s D. By comparing the genetic responses of larvae exposed to control and heat stress conditions, we aimed to elucidate the potential for rapid adaptation and evolutionary significance of genetic diversity in coral populations.

## Methods

### Coral and larvae

**Fig. 1:**
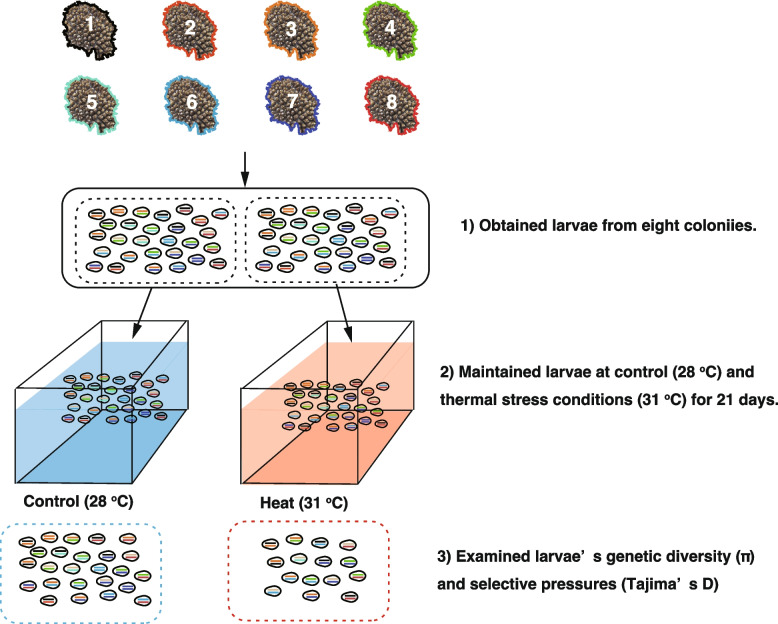
Schematic diagram of the study: larval production, aquarium experiments, and genetic analyses. **a** Larvae were obtained from eight *Acropora digitifera* colonies. **b** The pooled aposymbiotic larvae were kept in aquaria of control and heat conditions for 21 days. **c** Sixteen or ten larvae were kept and their DNA extracted to analyze genetic diversity, selective pressures with Tajima’s D values (balancing and positive selections with higher and lower Tajima’s D values, respectively), and codon evolution of coding sequences on these loci

Coral larvae were collected from eight *Acropora digitifera* colonies. Adult colonies were collected from the southern area of Sesoko Island (26.628989, 127.861517) and maintained under natural temperature (about 26 ^o^C) and light conditions for 7 days before the full moon in May 2023. We observed the colonies for signs of imminent spawning by checking their settings and the appearance of gamete bundles at approximately 20:30. When colonies were set for spawning, each colony was transferred to a 10 L bucket. Spawning started at approximately 22:20, and bundles were collected and mixed from eight colonies (Fig. 1). We roughly mixed equal numbers of bundles from each colony, although we did not count sperm concentrations. The bundles naturally broke apart without agitation.

The larvae were maintained at approximately 27 °C in buckets before the heat stress experiment, with room temperature kept stable to avoid significant fluctuations. While we did not monitor temperature variation in detail, the ambient conditions were controlled to prevent substantial deviations. Three days post-spawning, larvae were exposed to different temperature regimes: control (26.8 ± 10.27 °C, hourly mean ± SD) and heat (31.97 ± 0.19 °C, hourly mean ± SD). Filtered fresh seawater (pore size 1 μm) was continuously supplied to each aquarium at a mean rate of 150 mL/min, with overflow for water change. In each treatment group, three aquariums (2 L) were housed with 200 larvae each (*n* = 600 larvae per treatment) for 21 days (Fig. 1). To avoid overcrowding, 100 larvae were divided into tubes (two tubes per aquarium), each covered with a filter to prevent the larvae from escaping. The control temperature was set at 27 °C, reflecting the average sea-surface temperature during the experimental months, whereas the heat stress temperature was set to 31 °C, which, based on Degree Heating Weeks (DHW), is known to approach the bleaching threshold for *Acropora* corals at the study location [33]. Temperature elevation occurred gradually at a rate of 1 °C per day, facilitated by a heater (Everes, Micro save power Heater 75 W, Tokyo, Japan) regulated by a temperature controller (Power Thermo ET-308; Kotobuki, Osaka, Japan). Light intensity was measured using a light quantum sensor (Biosphere Instrument Inc., Q5L 2100, San Diego, CA, USA) twice daily (09:00 and 16:00), ranging between 120 and 150 µmol m⁻²s⁻¹. To minimize light-induced variations, the aquarium positions within each treatment were rotated biweekly. A temperature logger (HOBO Pendant Temperature/Light K Data Logger) was used to monitor the seawater temperature hourly.

In this study, no euthanasia or anesthesia was applied, as coral larvae (*A. digitifera*) do not require these methods. The larvae were sampled with a pipette to minimize stress and harm and preserved in ( e.g., ethanol or formalin). No anesthetic was necessary since coral larvae lack a nervous system capable of perceiving pain like vertebrates.

### Parameter measurements

Mortality, motility, and metamorphosis were assessed to determine the effects of heat stress on the ability of the larvae to disperse and settle. Mortality was defined as the percentage of static or translucent larvae observed after 24 h. Careful inspection (20× magnification) ensured that the larvae did not metamorphose or attach to tube walls. While motility and metamorphosis were not quantitatively measured, these parameters were monitored to minimize developmental stage variability and larval health variability. Live larvae that were cigar-like or spherical in shape were scored for motility at 20× magnification. We set up three replicates of control and heat conditions, and each tank generated one replicate of 200 larvae for mortality, motility, and metamorphosis each day when the incubations were completed.

### Larval and egg protein identification using LC-MS/MS

We used *A. digitifera* data from the NCBI database as reference information. We identified proteins in 7 days post fertilization (dpf) coral larvae at ambient temperature (approximately 100 larvae) and unfertilized eggs (approximately100 ~ 200 eggs) using liquid chromatography-tandem mass spectrometry (LC-MS/MS) as described by M Morita, N Hanahara, MM Teramoto and AI Tarigan [34]. Proteins from coral larvae were extracted, digested into peptides using trypsin, and analyzed by nano-liquid chromatography-tandem mass spectrometry (nano-LC-MS/MS) using a Thermo Fisher Scientific UltiMate 3000 RSLCnano system coupled with an Orbitrap Exploris 480, following standard procedures including desalination, peptide concentration adjustment, and MS data analysis with Scaffold DIA and Prosit, based on the *Acropora digitifera* protein database from NCBI. Identified proteins are shown in supplementary Table 1.

**Table 1:** Coding sequences under positive selection of the codons at loci with high or low Tajima's D values (95th or 5th percentile of control or heat-treated group)

**Scafold**	**Bp**	**Gene ID**	**LOC gene symbol**	**Annonation**	**Gene tended to be enriched**	**Larvae**	**Egg**	**model8**	**model8a**	**ΔlnL**	**P**
**Higher Tajima’s D (loci under balancing selection)**											
**Control**											
BLFC01000846.1	3280000	XM_015907420.1	LOC107341923	PREDICTED: Acropora digitifera probable pyridoxal 5'-phosphate synthase subunit PDX2 (LOC107341923), mRNA		*		-972.015	-975.278	6.526	<0.05
BLFC01000928.1	2190000	XM_015903984.1	LOC107338750	PREDICTED: Acropora digitifera RRP12-like protein (LOC107338750), mRNA		*		-4262.311	-4264.126	3.629	<0.05
BLFC01000174.1	70000	XM_015910308.1	LOC107344618	PREDICTED: Acropora digitifera uncharacterized LOC107344618 (LOC107344618), mRNA		*		-5096.159	-5097.464	2.612	0.106
BLFC01000330.1	185000	XM_015925259.1	LOC107358666	PREDICTED: Acropora digitifera uncharacterized LOC107358666 (LOC107358666), mRNA		*		-1807.258	-1809.811	5.107	<0.05
BLFC01000393.1	110000	XM_015915598.1	LOC107349451	PREDICTED: Acropora digitifera uncharacterized LOC107349451 (LOC107349451), mRNA	*	*		-4417.471	-4452.226	69.508	<0.001
BLFC01000505.1	250000	XM_015913829.1	LOC107347856	PREDICTED: Acropora digitifera transmembrane protein 62-like (LOC107347856), mRNA		*		-1668.563	-1678.819	20.511	<0.001
BLFC01000664.1	360000	XM_015921362.1	LOC107354869	PREDICTED: Acropora digitifera uncharacterized LOC107354869 (LOC107354869), mRNA		*		-9206.137	-9224.570	36.866	<0.001
BLFC01000404.1	1355000	XM_015901527.1	LOC107336449	PREDICTED: Acropora digitifera L-galactose dehydrogenase-like (LOC107336449), mRNA		*	*	-1373.631	-1375.597	3.933	<0.05
BLFC01000023.1	320000	XM_015901722.1	LOC107336651	PREDICTED: Acropora digitifera low-density lipoprotein receptor-related protein 4-like (LOC107336651), mRNA		*	*	-771.103	-773.461	4.716	<0.05
BLFC01000827.1	1320000	XM_015904761.1	LOC107339470	PREDICTED: Acropora digitifera SEC14 domain and spectrin repeat-containing protein 1-B-like (LOC107339470), mRNA		*	*	-3671.948	-3674.954	6.012	<0.05
BLFC01000366.1	135000	XM_015907330.1	LOC107341853	PREDICTED: Acropora digitifera serine/threonine-protein phosphatase 4 regulatory subunit 4-like (LOC107341853), mRNA	*	*	*	-1921.422	-1923.980	5.116	<0.05
BLFC01000460.1	340000	XM_015908520.1	LOC107342995	PREDICTED: Acropora digitifera FH1/FH2 domain-containing protein 3-like (LOC107342995), mRNA	*	*	*	-7472.228	-7491.145	37.833	<0.05
BLFC01000618.1	2430000	XM_015911077.1	LOC107345375	PREDICTED: Acropora digitifera ectopic P granules protein 5 homolog (LOC107345375), mRNA	*	*	*	-5890.780	-5896.450	11.339	<0.05
BLFC01000269.1	1085000	XM_015911798.1	LOC107346032	PREDICTED: Acropora digitifera SUN domain-containing ossification factor-like (LOC107346032), partial mRNA		*	*	-3148.075	-3161.406	26.662	<0.001
BLFC01000732.1	1870000	XM_015913505.1	LOC107347548	PREDICTED: Acropora digitifera BTB/POZ domain-containing protein KCTD5-like (LOC107347548), mRNA	*	*	*	-665.845	-668.937	6.182	<0.05
**Heat-Treated**											
BLFC01000348.1	5324000-5325000	XM_015900458.1	LOC107335438	LOW QUALITY PROTEIN: protein MAATS1-like (cillia & falgella associated protein 91 like)		*		-1601.662	-1605.482	7.639	0.022
BLFC01000426.1	300000	XM_015919325.1	LOC107353001	fat storage-inducing transmembrane protein 2-like	*	*		-1194.748	-1197.397	5.298	0.021
BLFC01000770.1	3815000	XM_015915728.1	LOC107349546	uncharacterized protein LOC107349546		*		-1138.717	-1142.439	7.444	0.024
BLFC01000846.1	3050000	XM_015913982.1	LOC107347984	baculoviral IAP repeat-containing protein 2-like	*	*		-1496.903	-1499.279	4.751	0.029
BLFC01000850.1	2400000	XM_015903983.1	LOC107338749	PREDICTED: Acropora digitifera dysferlin-like (LOC107338749), mRNA	*	*	*	-7403.717	-7418.893	30.351	<0.001
BLFC01000256.1	225000	XM_015904955.1	LOC107339639	PREDICTED: Acropora digitifera putative helicase MOV-10 (LOC107339639), mRNA		*	*	-467.971	-478.144	20.345	<0.001
BLFC01000766.1	740000	XM_015905474.1	LOC107340122	PREDICTED: Acropora digitifera filamin-A-like (LOC107340122), mRNA		*	*	-11699.636	-11704.082	8.892	0.012
BLFC01000632.1	1030000	XM_015905728.1	LOC107340374	PREDICTED: Acropora digitifera dynein heavy chain 1, axonemal-like (LOC107340374), partial mRNA		*	*	-8353.869	-8355.595	3.453	<0.05
BLFC01000734.1	230000	XM_015920562.1	LOC107354131	PREDICTED: Acropora digitifera serine/threonine-protein phosphatase 6 regulatory ankyrin repeat subunit B-like (LOC107354131), mRNA	*	*	*	-4372.007	-4449.523	155.033	<0.001
**Lower Tajima's D (loci under positive selection)**											
**Control**											
BLFC01000610.1	1000000	XM_015909580.1	LOC107343978	PREDICTED: Acropora digitifera uncharacterized LOC107343978 (LOC107343978), mRNA		*		-10607.993	-10612.005	8.025	0.005
BLFC01000610.1	1070000	XM_015923786.1	LOC107357143	PREDICTED: Acropora digitifera uncharacterized LOC107357143 (LOC107357143), mRNA		*		-6424.447	-6431.980	15.067	<0.001
BLFC01000648.1	260000	XM_015922026.1	LOC107355448	PREDICTED: Acropora digitifera spatacsin-like (LOC107355448), mRNA		*		-3389.585	-3393.335	7.500	0.006
BLFC01000701.1	600000	XM_015911256.1	LOC107345534	PREDICTED: Acropora digitifera mucin-5B-like (LOC107345534), mRNA		*		-9890.671	-9895.441	9.541	0.002
BLFC01000728.1	255000	XM_015917083.1	LOC107350832	PREDICTED: Acropora digitifera uncharacterized protein LOC107350832 (LOC107350832), mRNA		*		-1189.552	-1192.582	6.060	0.014
BLFC01000770.1	4175000	XM_015918631.1	LOC107352300	PREDICTED: Acropora digitifera transmembrane protein with metallophosphoesterase domain-like		*		-2332.660	-2335.345	5.370	0.020
BLFC01000850.1	1075000	XM_015924066.1	LOC107357425	PREDICTED: Acropora digitifera dimethylaniline monooxygenase [N-oxide-forming] 5-like	*	*		-3278.757	-3289.001	20.488	<0.001
BLFC01000161.1	1910000	XM_015917829.1	LOC107351535	PREDICTED: Acropora digitifera ceramide kinase-like		*		-3109.474	-3111.575	4.203	0.040
BLFC01000265.1	195000	XM_015911206.1	LOC107345488	PREDICTED: Acropora digitifera alanine aminotransferase 2-like isoform X3]		*		-2462.540	-2464.376	3.672	0.055
BLFC01000475.1	100000	XM_015916183.1	LOC107349988	PREDICTED: Acropora digitifera MAM and LDL-receptor class A domain-containing protein 2 (LOC107349988), mRNA		*		-1138.717	-1142.439	7.444	0.024
BLFC01000229.1	190000	XM_015894070.1	LOC107329378	PREDICTED: Acropora digitifera inositol monophosphatase 1-like (LOC107329378), mRNA		*	*	-553.892	-560.471	13.160	0.001
BLFC01000425.1	560000	XM_015898586.1	LOC107333735	PREDICTED: Acropora digitifera acetolactate synthase-like protein (LOC107333735), mRNA	*	*	*	-1896.409	-1898.555	4.292	0.038
BLFC01000057.1	1115000	XM_015900152.1	LOC107335166	PREDICTED: Acropora digitifera ubiquitin carboxyl-terminal hydrolase 24-like (LOC107335166), mRNA		*	*	-5308.371	-5309.897	3.052	0.217
BLFC01000023.1	1565000	XM_015909055.1	LOC107343486	PREDICTED: Acropora digitifera protein phosphatase 1 regulatory subunit 12A-like (LOC107343486), mRNA	*	*	*	-2847.095	-2854.443	14.695	0.001
BLFC01000166.1	2360000	XM_015910330.1	LOC107344640	PREDICTED: Acropora digitifera protein FAM102B-like (LOC107344640), mRNA		*	*	-690.610	-693.005	4.790	0.091
BLFC01000286.1	1235000	XM_015917796.1	LOC107351500	PREDICTED: Acropora digitifera dystonin-like (LOC107351500), mRNA		*	*	-212.673	-215.850	6.353	0.042
**Heat-Treated**											
BLFC01000324.1	1585000	XM_015902225.1	LOC107337105	BUD13 homolog isoform X1		*		-3061.759	-3071.002	18.486	<0.001
BLFC01000482.1	10000	XM_015924626.1	LOC107357989	pyruvate dehydrogenase phosphatase regulatory subunit, mitochondrial-like		*		-3389.585	-3393.335	7.500	0.006
BLFC01000439.1	3510000	XM_015893156.1	LOC107328419	cation-independent mannose-6-phosphate receptor-like	*	*	*	-8337.187	-8394.108	113.841	0.000
BLFC01000393.1	975000	XM_015913532.1	LOC107347569	calsequestrin-1-like	*	*	*	-1889.688	-1894.993	10.611	0.001
BLFC01000511.1	2185000	XM_015915947.1	LOC107349751	LOW QUALITY PROTEIN: vacuolar protein sorting-associated protein 41 homolog] [transl_except=(pos:1252..1254,aa:Other),(pos:2311..2313,aa:Other)	*	*	*	-3708.043	-3714.082	12.079	0.001
BLFC01000930.1	330000	XM_015919123.1	LOC107352810	GDP-fucose transporter 1-like	*	*	*	-1834.525	-1836.968	4.886	0.027
BLFC01000766.1	900000	XM_015922757.1	LOC107356117	importin subunit alpha-1-like	*	*	*	-2023.976	-2033.678	19.404	<0.001

### Genetic analyses to detect genetic diversity, selections, and codon evolution

Heat-exposed and control larvae were subjected to genome sequencing to examine the genetic diversity (π) and selective pressures (Tajima’s D) (Fig. 1). After experiments with control and heat-exposed larvae, larvae (16 larvae from the control condition and ten larvae from heat treated condition) were kept in 99.5% ethanol at −30 ^o^C, extracted DNA, and subjected to genome-wide amplicon sequencing, Gras-Di seq. To elute DNA, the larvae were treated with lysis buffer (150 mM KCl, Tween-20 0.1 (W/V)%, Triton X-100 0.1 (W/V)%, Tris-HCl (8.0)) with 1 (µg/ml) proteinase K (nacarai tesque, Kyoto, Japan) for 2.5 h at 55 ^o^C according to S Kitanobo, N Isomura, H Fukami, K Iwao and M Morita [35]. The supernatant, roughly having 50–200 ng/µL DNA, was used for the library preparation. We prepared a library according to M Furukawa, S Kitanobo, S Ohki, MM Teramoto, N Hanahara and M Morita [36]. Sequencing was conducted using a NovaSeq at Eurofin Co.

Due to unforeseen difficulties in isolating and preserving individual larvae, the number of samples successfully retained for genetic analysis was limited. Although we initially planned to analyze more than 20 larvae from each condition, some samples were lost during the preservation process in 96-well plates. As a result, 16 larvae from the control condition and 10 larvae from the heat-treated condition were included in the analyses.

The single nucleotide polymorphisms of the larvae were isolated from the reference genome of *A. digitifera*. First, fastq files were cleaned with fatsp [37] and mapped onto the reference genome (Adig_2.0, GCA_014634965.1) using BWA-mem2 [38] and samtools [39]. The reads of the sequences were from 199,840 to 8,506,516 (average 6290892). The vcf files were output from the bam files with population.pl implemented in Stacks [40]. The sequences’ depth on the reference varied (Supplementary Fig. 1), and the depth was about 15. The vcf files were filtered with Plink according to Hardy–Weinberg equilibrium (--HWE 0.000001), minor allele frequency (--MAF 0.01), and calling rates (--geno 0.25). Filtered files were transformed back into VCF format to calculate genetic diversity using π and Tajima’s D with VCFtools. The window size was set to 5000 bp due to the smaller scaffold size of the *A. digitifera* reference genome.

Non-calling sites were eliminated, and the values of the control and heat-treated groups were compared. Loci with the highest and lowest 5th percentiles of Tajima’s D and π values were identified, focusing on detecting selective pressures in the control or heat-treated group. Loci with higher Tajima’s D values were regarded as signals of balancing selection, whereas those with lower values indicated positive selection. Using the Basic Local Alignment Search Tool (BLAST), 1000-bp sequences were searched for before and after the loci with specifically high (95th percentile) or low (5th percentile) Tajima’s D values found only in the heat-treated or control groups. The best hit coding sequences (CDs) were isolated, and those functions were referred from Gene ontology data in NCBI database (GCF_000222465.1_Adig_1.1_gene_ontology.gaf.gz).

Diversity statistics (e.g., π, Tajima’s D) were calculated using vcftools, which excludes missing genotypes from the analysis. A higher missing genotype threshold (25%) was set, allowing more SNPs to be included in the analysis while acknowledging the tradeoff with missing data.

Although GO term assignment could not be performed due to the lack of GO terms registered in the NCBI gene bank database, GO analyses were conducted using the UniProt *Acropora digitifera* database with the GO_MWU tool [41]. For the analyses, we used the go.obo file (https://geneontology.org/docs/download-ontology/) and categorized coding sequences (CDs) according to Tajima’s D criteria under control and heat conditions. Lower Tajima’s D values imply positive selection, while higher values imply balancing selection. Based on this, we assigned CDs with higher or lower Tajima’s D values a score of 1, while others were assigned a score of 0. The GO_MWU tool utilizes the Mann-Whitney U (MWU) test to determine whether genes associated with a specific GO category are significantly enriched based on their position in a ranked list of genes. GO_MWU was then applied to analyze biological process (BP), molecular function (MF), and cellular component (CC) categories. Specific GO terms found under positive or balancing selection in control or heat conditions were analyzed using the Mann-Whitney U test. Despite the relatively small number of genes falling within these categories (20–50 genes) compared to the background as CDs encoding larvae proteins (6556 genes), we aimed to detect functional enrichment in these regions.

We then tested whether these genes underwent functional modifications during speciation. We examined the codon evolution of CDs using CodeML in the PAML package [42] in the loci with higher or lower Tajima’s D values, representing balancing or positive selection, respectively. Model 8 is a positive selection model for nonsynonymous mutations, and Model 8a is a neutral evolution model for nonsynonymous and synonymous mutations. We ran the two models and conducted a likelihood ratio test of these two models. The orthologs of the CDs were isolated with an orthoscope [43], a phylogenetic tree was constructed with RaxML [44], and the analyses were run with tree and nucleotide files according to M Furukawa, S Kitanobo, S Ohki, MM Teramoto, N Hanahara and M Morita [36].

### Statistics and reproducibility

Survival was treated as a proportion, with the number of surviving larvae divided by the total number of larvae in each tank. Survival was then plotted after Loess regression, and the Wilcoxon rank sum test was conducted to examine the differences between the control and heat conditions, with each tank considered as an independent replicate. Tajima’s D and π values were analyzed using a linear mixed model (LMM), with group set as a random effect. We used the lme4 package for this LMM analysis. To account for differences in sample size between the control and heat-stressed groups, we performed a random sampling analysis. For the control group, 10 individuals were randomly selected to match the sample size of the heat-stressed group, and this subsampling was repeated 100 times. Tajima’s D and π values were recalculated for each subsample, and the results were analyzed using a linear mixed model (LMM), with group set as a random effect. This approach ensured that the analysis was robust to sample size differences, and allowed us to examine whether the observed patterns in genetic diversity metrics were consistent across iterations. We used the lme4 package for the LMM analysis.

## Results

### Larval survival in heat and control conditions

**Fig. 2:**
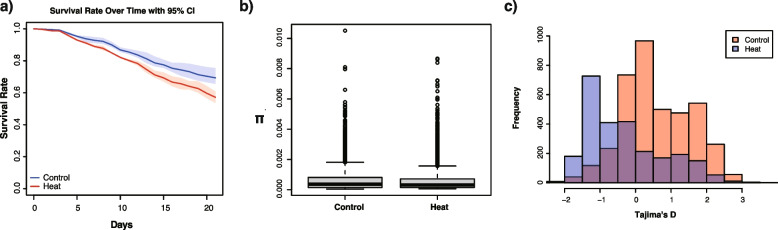
Larval survival, nucleotide diversity, and genetic variations in control and heat-exposed larvae. **a** Larval survival regressed according to the Loess methods, between the control and heat conditions. Three replicates of the regressed data and 95 confidence intervals are shown in the shaded region. Wilcoxon rank sum test shows differences between control and heat conditions (*P* < 0.05). **b** Nucleotide diversity is represented by π, and (**c**) genetic diversity is represented by Tajima’s D. Statistical comparisons between groups were performed using a generalized linear model, with a significant difference observed (*P* < 0.01)

The survival rate of the heat-treated larvae was significantly lower than that of the control group, with approximately 50% and 70% survival in the heat-treated and control groups, respectively (Fig. 2a; LOESS correlation of three replicates with 95% confidence intervals shown in red or blue shadows. Wilcoxon rank sum test, *P* < 0.05).

### Genetic diversity and potential selective pressures

The raw reads of the sequences were from 199,840 to 8,506,516 (average 6290892), and the average coverage was found to be 15.56x with a 95% confidence interval of (15.55, 15.57). However, the depth was not consistently high along the reference (supplementary Fig. 1; Supplementary information 1). This may cause the lower SNP number if we set up the –geno filtration to 0.05. Therefore, we set up geno 0.25 and MAF to 0.01 to call most genotypes. As a result of this filtration, we isolated 26,443 SNPs.

Nucleotide diversity (π) was not significantly different between the control and heat-stressed groups, indicating similar levels of genetic variation (Fig. 2b; linear mixed model (LMM, AIC = −72258, logLik = 36133, Chisq = 0.221, df = 1, *P* > 0.05). We re-ran the analyses by randomly subsampling 10 colonies from the control group to match the sample size of the heat-stressed group. This subsampling was repeated 100 times, and the results showed that π was also not significantly different between the groups in these subsampled comparisons (Supplementary information 2; *P* > 0.05). In contrast, the difference in Tajima’s D values between the two groups was consistently significantly different across analyses, even when controlling for sample size (Supplementary information 2), suggesting potential differences in selective pressures between the two groups (Fig. 2c; LMM, AIC = 18104, logLikelihood = −9092.9, Chisq = 14.9, df = 1, *P* < 0.001).

### Genomic regions under different types of selection

**Fig. 3:**
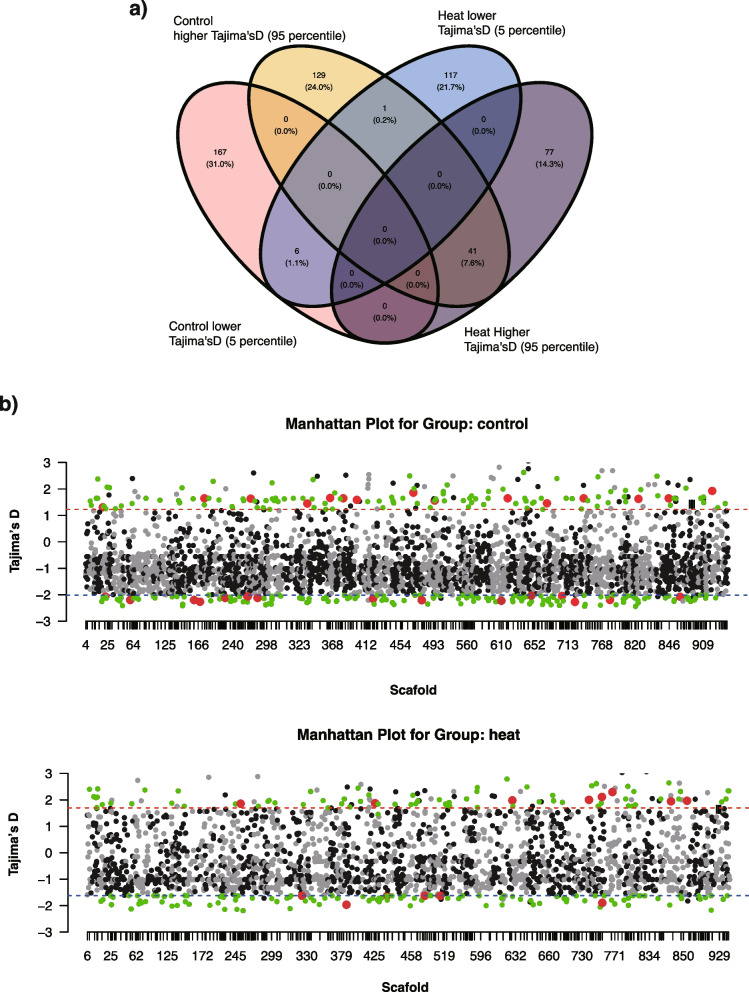
Venn diagram of genomic regions in higher or lower Tajima’s D values and Manhattan plot of those values. **a** Venn diagram of Tajima’s D values in lower (5 percentiles) or higher (95 percentiles) values in the control or heat-treated groups. **b** Plots of Tajima’s D values along with reference genomes of larvae from the control and heat-treated groups. Loci with lower (5th percentile) and higher (95th percentile) Tajima’s D values are indicated as positive or balancing selection, respectively. Specific genomic regions in control or heat-treated groups are marked in green dots. Red circles indicate CDs encoding larval proteins under positive selection of codon evolution

The loci identified as outliers for balancing selection (higher Tajima’s D values) and positive selection (lower Tajima’s D values) differed between the two groups (Fig. 3a; Table 1). The control group had 173 outlier regions with lower and 171 with higher Tajima’s D values, while the heat group had 118 outlier regions with lower and 124 with higher Tajima’s D values. Among the loci with higher Tajima’s D values, which may indicate balancing selection, 41 regions were shared between the two groups. In contrast, only 6 outliner regions with lower Tajima’s D values, which suggest positive selection, were shared. Most outliner regions were unique to each group, with 167 unique to control group and 117 unique to the heat-treated group) (Fig. 3a).

We searched coding sequences in these unique Tajima’s D regions. In the heat-treated group, 24 coding sequences (CDs) were identified in the genomic region under balancing selection, and 22 were identified in the regions under positive selection (Supplementary Table 1). Under the control condition, 39 and 45 CDs were detected in 95 and 5 percentile regions, respectively (Supplementary Table 2). As described above, the regions in the 95th to 100th percentiles of the highest Tajima’s D values in the control and heat-treated groups differed, with several coding sequences identified in these regions (Supplementary Table 2).

### Protein identification and functional annotation

**Fig. 4:**
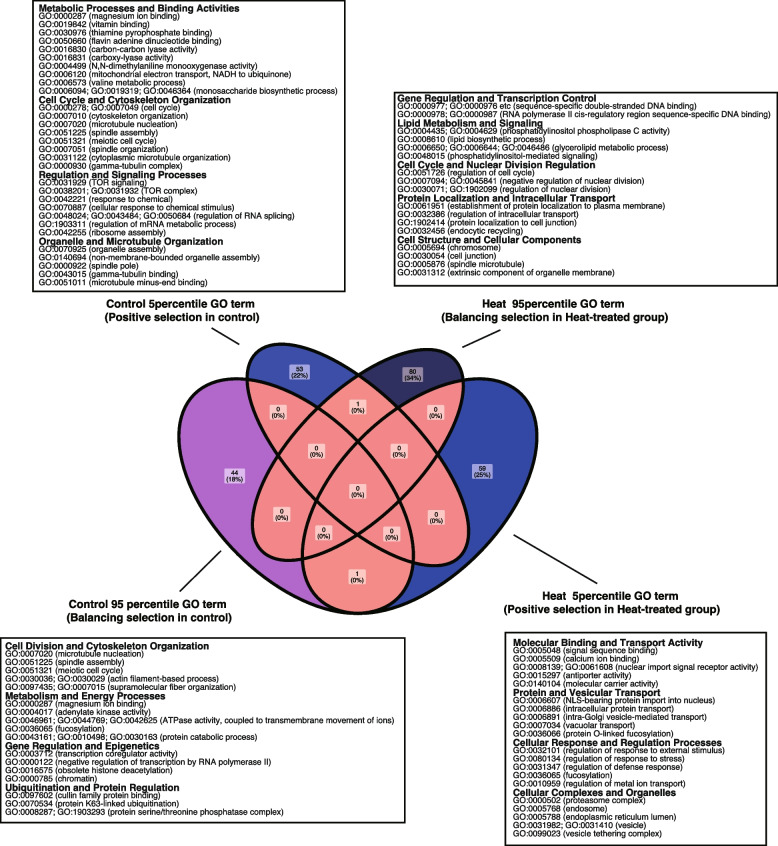
Venn diagram of GO terms of CDs encoding larval proteins in the genomic regions in higher or lower Tajima’s D values. Venn diagram of GO terms of CDs encoding the larval proteins in the genomic regions with lower (5 percentiles) or higher (95 percentiles) Tajima’s D values in the control or thermal-stressed groups. The predicted cellular functions from GO terms were indicated in each panel

Protein identification of coral larvae using liquid chromatography tandem mass spectroscopy (LC-MS/MS) indicated that some CDs encoded proteins in the larvae (Fig. 3a; Table 1, and Supplementary Tables 1, and 2). Among the coding sequences (CDs) encoding proteins in the larvae, we identified proteins not common with those in unfertilized eggs, representing proteins expressed after fertilization. Many identified CDs encode uncharacterized proteins, while others are functionally annotated with Gene Ontology (GO) terms. We performed enrichment analyses on the CDs from each group and examined whether the GO terms associated with the CDs in each group were shared or distinct. Most GO terms in each group were unique to their respective groups. Furthermore, within each group, the GO terms associated with genes located in regions of high Tajima’s D values (indicative of balancing selection) did not overlap with those associated with genes in outliner regions of low Tajima’s D values (indicative of positive selection). Similarly, in the heat-treated group, the GO terms associated with high and low Tajima’s D regions were also distinct. This lack of overlap, both within and between groups, highlights condition-specific selection pressures and further emphasizes the functional differences in genes under balancing and positive selection (Fig. 4; Supplementary data 1).

In control group, many of the GO terms are cell cycles and metabolisms (Fig. 4; Supplementary data 1). In the heat-treated group, lipid metabolisms and stress responses. Although the enrichment analyses did not reveal significantly enriched genes when adjusted P values were referred to, it is important to note that the target gene set was relatively small, consisting of fewer than 50 genes, compared to the background gene set of larval protein encoding CDs (6,556). We also considered non-adjusted p-values, which indicated significant results. Specifically, group-specific GO terms were significantly enriched (Control 95th percentile: 23/45, Control 5th percentile: 20/54, Heat 95th percentile: 15/81, Heat 5th percentile: 18/60). GO terms tended to be enriched were uncommon among the group (Supplementary Fig. 2; Supplementary Data 1).

### Selection pressures and codon evolution

Our CodeML analysis of the coding sequences (CDs) encoding larval proteins focused on identifying codons undergoing positive selection during acroporid speciation. This analysis revealed that most genes in outlier regions, based on Tajima’s D values, are under positive selection at the codon level (Fig. 3b - red dot; Table 1; Supplementary Table 2). In control conditions, 14 of 15 genes in regions with higher Tajima’s D values (indicative of balancing selection) and 14 of 14 genes in regions with lower Tajima’s D values (indicative of positive selection) exhibited positive selection at the codon level (Table 1). In heat-treated conditions, 9 of 9 genes in regions with higher Tajima’s D values and 7 of 7 genes in regions with lower Tajima’s D values showed positive selection at the codon level (Table 1).

Our results also suggest that nucleotide-level balancing selection (regions with higher Tajima’s D values) may coexist with codon-level positive selection in certain genes. This coexistence could facilitate the maintenance of advantageous alleles within these regions. Additionally, all genes with enriched GO terms were found to be under positive selection at the codon level (Table 1).

## Discussion

In this study, we examined how the genetic diversity of coral hosts changes in response to heat stress in larvae before they establish a symbiotic relationship. Genetic diversity resulting from sexual reproduction is crucial for the adaptability and long-term survival of populations and provides a reservoir for advantageous genetic variation under fluctuating water temperature conditions. Selective pressures, as indicated by Tajima’s D values, differed between the control and heat stress groups, whereas nucleotide diversity (π) did not differ significantly. This suggests that thermal stress can rapidly influence selective pressure on larvae during the experimental period, with both control and heat stress conditions showing evidence of selective pressures, as indicated by Tajima’s D values. While we recognize that positive Tajima’s D values can also result from demographic history, such as population contraction, our study focuses on short-term selective pressures arising during the experimental period. The observed differences in Tajima’s D values likely reflect condition-specific selection pressures rather than long-term population dynamics. Additionally, it is important to note that genetic drift or other stochastic processes may also contribute to variations in Tajima’s D values. Our approach thus highlights how experimental conditions can influence selection dynamics over a short time scale, differentiating this from broader evolutionary processes.

Although Tajima’s D values are often used to indicate changes in population size, those values are also influenced by other factors such as selection, genetic drift, and demographic events. It is important to note that while global values of Tajima’s D and nucleotide diversity provide an overall view of population dynamics, they do not necessarily align with the patterns observed in outlier loci. Outlier loci indicate regions under strong selective pressures, which may differ from the global genomic patterns. For example, in this study, outlier loci with high Tajima’s D values (indicative of balancing selection) and low Tajima’s D values (indicative of positive selection) were predominantly unique to each experimental condition. Specifically, we identified 41 outlier regions with high Tajima’s D values shared between the control and heat-treated groups, while only 6 outlier regions with low Tajima’s D values were shared. This disparity suggests that selective pressures, both balancing and positive, are highly condition-specific, emphasizing the importance of distinguishing between global and localized patterns when interpreting genomic data. Our study thus differentiates between global genomic measures and localized selection effects to provide a more nuanced interpretation of the data.

Larval dispersal in coral varies with water temperature over short periods, suggesting that selective pressures on larvae may shift depending on environmental conditions. For instance, higher water temperatures are reported to shorten the larval period [15], potentially limiting the time available for settlement and exposing larvae to different selective pressures. Multiple factors, including egg size, influence the timing of larval settlement competency, and this timing does not follow a straightforward pattern, as discussed by Randall et al., [45]. Our study found that genomic regions with higher or lower Tajima’s D values differed between the control and heat stress groups. Additionally, genes related to metabolism and stress responses, inferred from GO terms, were identified in higher Tajima’s D regions under both control and heat conditions (Fig. 4). While no significantly enriched GO terms were detected after corrections for multiple comparison, the metabolism-related GO terms showed a tendency for enrichment, as indicated by p-values before corrections for multiple comparisons.

The number of colonies participating in synchronous spawning plays a crucial role in shaping the genetic diversity of subsequent generations. In other words, promiscuous fertilization of colonies results in diverse larvae, and changes in genetic diversity at the larval stage might indicate natural selection. Notably, bleaching events reduce the number of released gametes owing to a decline in colony numbers [46] and a decline in gamete production [47], potentially accelerating the decrease in the genetic diversity of coral larvae. While our study did not detect a significant difference in nucleotide diversity between the heat and control groups, we identified specific loci under selection pressures related to metabolism and stress responses. These results suggest that even in the absence of large-scale changes in overall nucleotide diversity, particular genes may be under selective pressure, highlighting the importance of maintaining genetic diversity to ensure that such critical genomic regions can be under selective pressure in response to environmental stressors.

Our analysis suggests that selections may act on genes related to cellular functions, as indicated by GO terms such as metabolisms. Metabolic processes play a critical role in supporting energy demands during stress, including heat stress [48, 49], which could explain why genes involved in these pathways might be under balancing selection. Balancing selection maintains genetic diversity at these loci, potentially providing populations with the flexibility to adapt to fluctuating environments, including episodic thermal stress. For instance, co-chaperone sacsin is reported to be under selection, is thought to contribute to thermal tolerance by assisting in protein folding under heat stress conditions [21]. Although we did not identify sacsin under selections (High or Low Tajima’s D), genes involved in key metabolism-related pathways were identified (Fig. 4). These pathways are predicted to be crucial for maintaining larval survivorship and cellular homeostasis during environmental stress. Interestingly, the functions predicted from GO terms differed between the control and heat-stressed groups, suggesting condition-specific selective pressures. Future research is needed to elucidate how balancing selection on these metabolic pathways might contribute to long-term thermal tolerance in adult colonies.

Our individual larvae sequencing revealed that the surviving larvae had specific genetically divergent loci between the control and heat-stressed groups, particularly those related to metabolism and ontogenic processes, and were larva-composing proteins. Although we only functionally quantified proteins in control larvae, genes for lipid metabolism, such as fat storage-inducing transmembrane protein 2-like, were under balancing selection, as indicated by higher Tajima’s D values, in heat-stressed larvae. Lipids are crucial for coral larval metabolism prior to symbiont acquisition, and metabolic rates of lipids are rapid in non-symbiotic larvae [9], highlighting the role of lipid metabolism in the survival of coral larvae subjected to heat stress in this study. While lipid profiles vary depending on the location and origin of the colonies [50], and lipid depletion did not affect larval survival under heat stress [51], lipid remain a critical energy reserve for larvae before establishing a symbiotic relationship. Unique outlier genomic regions were identified in both the control and heat-treated groups, indicating condition-specific selective pressures (Fig. 3a). However, the genetic divergence of the enzymes involved in lipid metabolism needs to be clarified, as they are associated with lipid consumption and heat tolerance.

Our study highlights that some genes identified under selection during speciation were also found undergoing selection in this study (Table 1). For instance, genes associated with specific functions, such as metabolism, which were under positive selection at the codon level during Acroporid speciation, were observed in both control and heat-stressed larvae. In the heat-treated group, these genes were linked to GO terms related to metabolism, indicating their potential role in facilitating adaptation to elevated water temperatures. This suggests that genetic diversity shaped by past speciation events contributes to adaptation to contemporary environmental stressors. In contrast, genes identified in the control group were associated with GO terms related to developmental processes (Table 1; Fig. 4), reflecting their role in maintaining baseline physiological functions under stable conditions.

Outlier loci differed markedly between the control and heat-stressed groups, indicating condition-specific selective pressures. Loci unique to the control group may reflect selection in stable environments or represent demographic history, such as past population contractions or expansions. In the heat-treated group, unique loci were associated with GO terms related to stress response pathways, underscoring the role of selective pressures driven by elevated water temperatures. These findings suggest that selective pressures are influenced by long-term evolutionary processes and contemporary environmental conditions, such as thermal stress, which drive localized adaptation. This dual role of balancing and positive selection highlights the complex genetic mechanisms underpinning thermal tolerance and emphasizes the importance of genetic diversity in enabling coral larvae to survive under fluctuating environmental conditions.

The heat stress response in the coral occurs via complex interactions between the host and holobionts; however, we only focused on aposymbiotic larvae to examine the selective pressures of heat stress on the host genome independently of symbiotic influences. Selective pressures may act differently throughout the coral’s life cycles, with symbiotic larvae potentially experiencing additional or distinct mechanisms due to the presence of symbiotic algae [52]. Physiological responses, including gene expression changes related to immune response and stress tolerance, have been shown to vary between apo- and symbiotic larvae [52]. Once the symbiotic relationship is established, selection may continue to shape larval survival and adaptation through various environmental pressures. In various genera, the larvae from heat-tolerant colonies are reported to be more tolerant in *Platygyra daedalea* [6], while another study on *Montipora capitata* found no clear relationship between colony heat tolerance and larval heat tolerance [18]. This inconsistency likely results from the complexity of heat tolerance mechanisms, involving interactions between the symbiotic algae and the coral host [53]. Although the symbiotic relationship appears to influence selective pressures as part of hologenome, most genetic divergences have been observed in the host genome, with minimal differences in the symbiotic communities among divergent populations [31].

This study identified specific genomic regions under selection in response to heat stress in *A. digitifera* larvae. Our genomic analyses provide insights into the genetic basis of heat tolerance, revealing potential candidate genes encoding larval proteins that may play a role in the adaptation of coral larvae to thermal stress. Additionally, genomic regions distinct from those under heat-treated conditions were predicted to be under selection in control groups. While we did not directly assess phenotypic outcomes, these findings contribute to our understanding of how corals respond to increasing ocean temperatures genetically. However, it is essential to note that the selection pressure applied in this study was moderate, which may have resulted in subtle genetic changes that did not lead to significant GO enrichment for specific pathways. This highlights the need for future studies to apply more intense or prolonged heat stress treatments to fully capture the genetic basis of adaptation. Furthermore, our study emphasizes the need for further research into the role these identified genes may play in the long-term survival of coral populations.

This study identified specific genomic regions under selection in response to heat stress in *A. digitifera* larvae. Our genomic analyses provide insights into the genetic basis of heat tolerance, revealing potential candidate genes encoding larval proteins that may play a role in the adaptation of coral larvae to thermal stress. Additionally, genomic regions distinct from those under heat-treated conditions were predicted to be under selection in control groups. While we did not directly assess phenotypic outcomes, these findings contribute to our understanding of how corals respond to increasing ocean temperatures genetically. Furthermore, our subsampling analysis revealed that nucleotide diversity (π) was also not significantly different in the control group after adjusting the sample size to match the heat-stress group, Tajima’s D values remained consistently different between the two groups across all iterations. This finding underscores the robustness of Tajima’s D as an indicator of selective pressures, even when sample size differences are accounted for. The consistency in Tajima’s D suggests that the observed differences reflect true biological processes, likely driven by condition-specific selective pressures, rather than artifacts introduced by sample size variability. These results emphasize the importance of considering both global and localized genomic patterns to disentangle the complex interplay between selection and genetic drift under varying environmental conditions. However, it is essential to note that the selection pressure applied in this study was moderate, which may have resulted in subtle genetic changes that did not lead to significant GO enrichment for specific pathways. This highlights the need for future studies to apply more intense or prolonged heat stress treatments to fully capture the genetic basis of adaptation. Furthermore, our study emphasizes the need for further research into the role these identified genes may play in the long-term survival of coral populations.

## Supplementary Information

Supplementary Material 1.

Supplementary Material 2.

Supplementary Material 3.

Supplementary Material 4.

Supplementary Material 5.

Supplementary Material 6.

Supplementary Material 7.

## Data Availability

All sequence data generated and analyzed during this study have been deposited in GenBank under the project accession number PRJNA1175841. Scripts used for the analyses are available in the GitHub repository at (https://github.com/Masaya0606/Aditifera_larvae). Additional datasets and materials are provided in the supplementary materials of this manuscript.
