# Supplementary material for: Slight thermal stress exerts genetic diversity selection at coral (Acropora digitifera) larval stages
Source: BMC Genomics. 2025 Jan 14;26:36. doi: 10.1186/s12864-024-11194-1 (PMC11730148; doi:10.1186/s12864-024-11194-1)
Supplement: Supplementary file 5 — Supplementary Material 5. [file 12864_2024_11194_MOESM5_ESM.docx]

**Supplementary information 1. Coverage of each sample**

Sample: 71_8

Mean Coverage: 13.49079938453698

95% Confidence Interval: (13.457620085592907, 13.523978683481051)

Sample: 72_8

Mean Coverage: 20.84924242309805

95% Confidence Interval: (20.69333561642274, 21.005149229773362)

Sample: 73_8

Mean Coverage: 12.893547939767885

95% Confidence Interval: (12.86918478632667, 12.9179110932091)

Sample: 74_8

Mean Coverage: 16.96021221595524

95% Confidence Interval: (16.905568944799295, 17.014855487111188)

Sample: 75_8

Mean Coverage: 17.34722284184698

95% Confidence Interval: (17.292248213620468, 17.40219747007349)

Sample: 76_8

Mean Coverage: 14.567869886552483

95% Confidence Interval: (14.532150233108348, 14.603589539996618)

Sample: 77_8

Mean Coverage: 14.152883475467908

95% Confidence Interval: (14.122723414426346, 14.18304353650947)

Sample: 78_8

Mean Coverage: 14.080084713146313

95% Confidence Interval: (14.047707469421457, 14.112461956871169)

Sample: 79_9

Mean Coverage: 16.00550486450607

95% Confidence Interval: (15.863157309929916, 16.147852419082223)

Sample: 80_9

Mean Coverage: 23.339150452283388

95% Confidence Interval: (23.055141645560706, 23.62315925900607)

Sample: 81_9

Mean Coverage: 15.917349437935199

95% Confidence Interval: (15.881649108767922, 15.953049767102476)

Sample: 82_9

Mean Coverage: 12.647670705500538

95% Confidence Interval: (12.62883192617429, 12.666509484826786)

Sample: 83_9

Mean Coverage: 17.127088499908506

95% Confidence Interval: (17.091047218555804, 17.16312978126121)

Sample: 84_9

Mean Coverage: 15.820758350890602

95% Confidence Interval: (15.790634892090507, 15.850881809690696)

Sample: 85_9

Mean Coverage: 15.901576178354421

95% Confidence Interval: (15.869972576162178, 15.933179780546665)

Sample: 86_9

Mean Coverage: 21.492985751278944

95% Confidence Interval: (21.306078938392865, 21.679892564165023)

Sample: 87_10

Mean Coverage: 14.978754037180991

95% Confidence Interval: (14.93483182785855, 15.022676246503432)

Sample: 88_10

Mean Coverage: 14.324706550346287

95% Confidence Interval: (14.29868722039387, 14.350725880298704)

Sample: 89_10

Mean Coverage: 16.900083580668458

95% Confidence Interval: (16.866798796350043, 16.933368364986872)

Sample: 90_10

Mean Coverage: 22.76921311766014

95% Confidence Interval: (22.646965110716447, 22.891461124603833)

Sample: 91_10

Mean Coverage: 18.44325726110594

95% Confidence Interval: (18.404601244760958, 18.481913277450925)

Sample: 92_10

Mean Coverage: 17.079627298686887

95% Confidence Interval: (17.045803909762196, 17.11345068761158)

Sample: 93_10

Mean Coverage: 24.257672654365706

95% Confidence Interval: (24.141620143479958, 24.373725165251454)

Sample: 94_10

Mean Coverage: 18.705745392879518

95% Confidence Interval: (18.641161326648646, 18.77032945911039)

Sample: 95_11

Mean Coverage: 14.980192500570356

95% Confidence Interval: (14.952762755683223, 15.007622245457489)

Sample: 96_11

Mean Coverage: 17.034566195095618

95% Confidence Interval: (17.00045819179299, 17.068674198398245)
